# From Skin to Kidneys: Cutaneous Clues of Renal Disease in Children

**DOI:** 10.5826/dpc.1004a95

**Published:** 2020-10-26

**Authors:** Mario Diplomatico, Pierluigi Marzuillo, Angela La Manna, Andrea Apicella, Stefano Guarino, Vincenzo Piccolo

**Affiliations:** 1 General and Specialized Surgery for Women and Children, University of Campania Luigi Vanvitelli, Naples, Italy; 2 Emergency and ICU Departments, AORN Santobono-Pausilipon, Naples, Italy; 3 Dermatology Unit, University of Campania Luigi Vanvitelli, Naples, Italy

**Keywords:** pediatrics, dermatology, nephrology, vasculitides, syndromes

## Abstract

**Background:**

The skin is often seen as a world apart, but not rarely do cutaneous manifestations reveal signs of systemic disease.

**Objectives:**

The aim of this review is to include in one paper all the possible correlations between nephrological and dermatological manifestations of the same disease in pediatric patients while also keeping in mind that in apparent exclusively dermatological diseases there can be nephrological manifestations as part of the same disorder and vice versa.

**Methods:**

We searched on PubMed for a possible link between skin and kidney matching the following terms and correlated MeSH terms: dermatology, skin, kidney, renal disease, nephrology, pediatrics, child, childhood, vasculitis, and cancer. We selected only articles reporting a link between nephrology and dermatology in pediatrics, and they are all included in this comprehensive review.

**Results:**

Kawasaki disease, Henoch-Schönlein purpura, systemic lupus erythematosus, Dent disease, subcutaneous fat necrosis, Langerhans cell histiocytosis, renal cell carcinoma, non-Hodgkin lymphoma, tuberous sclerosis complex and syndromes with increased risk for Wilms tumor, Fabry disease, nail-patella syndrome, neurofibromatosis type 1, Beckwith-Wiedemann syndrome, Adams-Oliver syndrome 1, Apert syndrome, Fanconi pancytopenia syndrome, Pallister-Hall syndrome, and Fanconi pancytopenia syndrome are all conditions in which there can be both nephrological and dermatological manifestations in children.

**Conclusions:**

We could not find any reports that focused attention on the link between nephrological and dermatological manifestations of the same disease in children. It is also important for clinicians to keep in mind that in what may appear to be an exclusively dermatological disease, there can be nephrological manifestations as part of the same disorder and vice versa.

## Introduction

The skin is primarily involved in many diseases, and some cutaneous manifestations can also reveal signs of an internal disease. Renal disease is revealed by the changing amount, concentration, elements, and color of the urine, but sometimes it can be suspected based on cutaneous manifestations. What about the possibility of recognizing or suspecting renal diseases by skin manifestations in pediatric patients? The aim of the present review is to examine cutaneous involvement with nephrological diseases, to explore the importance of the cutaneous manifestations as a clue to kidney disease, and to put the attention on their possible link.

## Search Strategy

We searched PubMed for a possible link between skin and kidney using these search strategies: (“Kidney”[MeSH] AND “Dermatology”[MeSH] AND “Pediatrics”[MeSH]), (“Kidney”[MeSH] AND “Dermatology”[MeSH] AND “Child”[MeSH]), (“Kidney”[MeSH] AND “Dermatology”[ MeSH] AND “Childhood”), (“Nephrology”[MeSH] AND “Dermatology”[MeSH] AND “Child”[MeSH]), (“Nephrology”[MeSH] AND “Child”[MeSH] AND “Skin”[MeSH]), (“Kidney Diseases”[MeSH] AND “Dermatology”[ MeSH] AND “Child”[MeSH]), (“Kidney Diseases”[MeSH] AND “Dermatology”[MeSH] AND “Pediatrics”[MeSH]), (“Vasculitis”[MeSH] AND “Dermatology”[ MeSH] AND “Pediatrics”[MeSH]), (“Vasculitis”[ MeSH] AND “Kidney”[MeSH] AND “Pediatrics”[ MeSH]), (“Neoplasms”[MeSH] AND “Dermatology”[ MeSH] AND “Pediatrics”[MeSH]), (“Neoplasms”[ MeSH] AND “Kidney”[MeSH] AND “Pediatrics”[ MeSH]). We selected only articles that reported a link between nephrology and dermatology in pediatrics, and they are all included in this comprehensive review.

## Vasculitis

Vasculitis is different from systemic lupus erythematosus (SLE) in that inflammation of blood vessels may occur as a primary process, and because of multisystemic involvement, many subspecialties are involved in its diagnosis.

In pediatrics, the most common primary medium to small vessel vasculitides in childhood are Kawasaki disease (KD) and Henoch-Schönlein purpura (HSP). KD is a febrile disease associated with polymorphous exanthema, changes in extremities (erythema of the palms and soles, indurative edema of the hands and feet, swelling, and fissuring between the nails and the tips of the fingers), bilateral bulbar conjunctivitis without exudate, lip and oral cavity involvement, and cervical lymphadenopathy. Notably, it is always associated with the risk of cardiac damage. As recently reported by Chuang et al, renal involvement is underestimated, and they found it in 52% of the cases with a significant association between acute kidney injury (AKI) and age and alanine transaminase (ALT) level: the younger the age and/or the higher the ALT value, the more important it is to check renal function during both acute and convalescent phases. They also found that sterile pyuria was more prevalent in KD patients with AKI than in the non-AKI group (P < 0.01). Although they did not find a significant correlation at the multivariate logistic regression (P = 0.72), recent studies are frequently supporting the involvement of kidney, bladder, and urethra in its formation [1,2].

Henoch-Schönlein purpura is an IgA vasculitis characterized by lower extremity purpura that is variably associated with abdominal, joint and renal involvement. In contrast to KD, the risk of renal involvement (hematuria and proteinuria within or beyond nephrotic range) in patients affected by HSP increases with age: children older than 8 years have a 2.7-fold higher risk of nephritis [3].

The most common primary large vessel vasculitis in childhood is Takayasu arteritis, even though it is predominantly reported in the second and third decades of life, most likely due to its subtle onset that contributes to a delayed diagnosis. It is mainly characterized by constitutional symptoms, alteration of peripheral pulses, hypertension (due to the narrowing of one or both renal arteries, often overlooked in children), and arthralgias, but it may also present with skin involvement, such as rashes or nodules resembling pyoderma gangrenosum or erythema nodosum [4].

As a key point, we suggest checking renal involvement in almost every vasculitis.

## Systemic Lupus Erythematosus

Systemic lupus erythematosus (SLE) is a chronic autoimmune inflammatory disease affecting many organs. The manifestations in children are the same as in adults, but children are more often affected by a severe organ involvement as a presenting manifestation, primarily nephropathy and hematological involvement [5]. Cutaneous manifestations (some of which are included in the diagnostic criteria) are extremely common in SLE, as they present as the first sign in up to 25% of cases and are classified into specific and non-specific lupus manifestations. Lupus-specific manifestations are acute (malar rash and photosensitive lupus rash), subacute (maculopapular rash), chronic (discoid rash), whereas non-specific ones are mucosal ulcers, non-scarring alopecia, and vascular abnormalities (periungual erythema, livedo reticularis, Raynaud phenomenon, vasculitis) [6]. Variable degrees of renal involvement are present in up to 70% of children affected by SLE. Lupus nephritis is defined by kidney biopsy in 6 classes and can be present without any other sign of SLE [7]. Lupus nephritis is not the only way in which SLE can involve the kidney. Other forms of renal involvement are: tubulointerstitial nephritis (strong correlation with the prognosis for hypertension, elevation of plasma creatinine concentration, and progressive clinical course); vascular disease (with deposition of immune deposits in the glomeruli, thrombotic microangiopathy, and atherosclerosis); glomerular podocytopathy (independent of immune complex deposition); and collapsing glomerulosclerosis (similar to HIV-associated nephropathy) [8–11].

If SLE is suspected, screening for renal involvement is suggested, ie, a urinalysis to rule out the presence of hematuria and proteinuria.

## Dent Disease

Dent disease is a heterogeneous X-linked recessive disorder that, similarly to Fanconi syndrome, presents with low molecular weight proteinuria, hypercalciuria, nephrocalcinosis, metabolic bone disease, and progressive renal failure. Up until now, due to its heterogeneity, only 2 genes have been identified: *CLCN5* (Dent disease type 1–60% of cases) and *OCRL1* (Dent disease type 2–15% of cases). The remaining percentage of disease remains genetically unexplained, as there have been reported cases of patients with Dent disease phenotype without mutations in these 2 genes [12,13]. No association between skin and Dent disease type 2 (DD-2) before 2018 was reported, although it has been reported with Lowe syndrome, namely, oculocerebrorenal syndrome; secondary to a mutation in the same *OCRL1* gene; and sometimes presents with eruptive hair vellus hair cysts, trichoepithelioma, and excessive skin folds [14]. Marzuillo et al reported the possible association between DD-2 and hidradenitis suppurativa. Hidradenitis suppurativa has a prevalence of 1% in the general population with an average age onset in the second to third decade of life but a prevalence of 80% in patients affected by DD-2: all males, with mean age of onset of 13 years. As postulated, a possible explanation could be an increased susceptibility to staphylococcal cutaneous infections and a stimulation of cutaneous inflammation associated with impairment of the junctional components due to mutations in *OCRL1* gene that drastically reduce (<10%) inositol polyphosphate 5-phosphatase (*OCRL1*) activity [15]. Therefore, Dent disease should be taken into consideration when evaluating a male child or a young boy with hidradenitis suppurativa and proximal tubule dysfunction with low molecular weight proteinuria and hypercalciuria, nephrolithiasis, nephrocalcinosis, progressive renal failure, and intellectual disability.

## Fabry Disease

Fabry disease is an X-linked metabolic disorder due to a deficiency of lysosomal α-galactosidase A. This deficiency leads to accumulation of glycosphingolipids in the whole body, including the heart and peripheral and autonomic nervous systems later in life. Onset of symptoms seems to occur earlier in males than in females (mean age 10.9 vs 22.6 years). Children diagnosed with Fabry disease can develop proteinuria, anhidrosis, and angiokeratoma. Early recognition is important because increasing evidence suggests that enzyme replacement treatment is most beneficial in terms of clinical outcome when initiated early in the disease process [16].

## Subcutaneous Fat Necrosis

Until the establishment of protocols for therapeutic neonatal hypothermia, a documented complication was subcutaneous fat necrosis (SFN). SFN is a granulomatous disorder associated with perinatal asphyxia, trauma, or prolonged exposure to cold, that presents with subcutaneous indurated purple-colored nodules associated with hypercalcemia (probably because of the damage to the fat tissue). Although not clearly understood, the mechanism of hypercalcemia in SFN may be due to the damage to fat tissue that causes necrosis, increased production of 1.25-dihydroxyvitamin D by macrophages, increased calcium absorption from the gastrointestinal tract, and osteoclast activation by prostaglandins. Severe hypercalcemia (calcium level >3 mmol/L) is a life-threatening condition at any age, has a mortality rate of up to 15% in SFN, and it is associated with kidney injury, with or without nephrocalcinosis, as well as with cardiac complications [17]. In the case of severe hypercalcemia a urinalysis and kidney ultrasounds are suggested.

## Langerhans Cell Histiocytosis

Langerhans cell histiocytosis (LCH) is a rare disease characterized with various presentations. The most commonly involved sites are bones, skin, lymph nodes, pituitary gland, liver, lung, and spleen, and the dissemination affects the prognosis and treatment. Histopathology shows monoclonal Langerhans cells together with lymphocytes, eosinophilic granulocytes and non-dendritic histiocytes. This disease usually presents on the skin with brown or purplish papules and an eczematous rash (often misdiagnosed as “cradle cap” on the scalp). Renal involvement is rarely reported in the literature but is characterized by a variable infiltration of glomeruli or interstitium, and presents as nephrotic syndrome or interstitial nephritis [18]. More frequently, polyuria and polydipsia can be the nephrological signs of pituitary gland involvement causing central diabetes insipidus [19].

## Renal Cell Carcinoma

Renal cell carcinoma is extremely rare in children, but is more common in adolescents older than 15 years of age. The classic triad of hematuria, flank pain, and palpable abdominal mass is present in less than 10% of adult cases; the diagnosis is often incidental. The skin may be involved by flushing because of prostaglandins production [20].

## Non-Hodgkin Lymphoma

Non-Hodgkin lymphoma is a tumor originating from lymphoid tissues, mainly lymph nodes, that in about 30% of cases presents with extranodal involvement at the diagnosis. The most commonly involved sites are skin, bone marrow, central nervous system, gastrointestinal and genitourinary tract, thyroid, and sinuses. The skin is the second most common extranodal localization of disease presenting with rashes or lesions that should always be biopsied. The possible renal involvement (up to 14% of cases) includes enlarged kidneys, ureteral obstruction (due to retroperitoneal disease), tubular dysfunction (due to acute uric acid nephropathy), and renal failure [21].

## Other Syndromes, Malformations and Genetic Disorders

Wilms tumor (WT) is rarely diagnosed in the neonate, but it is the most common renal cancer in childhood (95% of cases). In approximately 10% of cases, it is part of syndromes such as WAGR, Beckwith-Weidemann, and Denys-Drash. Certain overgrowth conditions associated with capillary malformation and hemihypertrophy or macrocephaly-capillary malformation also have an increased risk for WT; in these cases, a ultrasonographic screening for WT is necessary [22].

Tuberous sclerosis complex is an autosomal dominant genetic disorder (*TSC1* or *TSC2* genes) with an incidence of 1:5000–10000 that involves many organ systems. Reported skin lesions are hypopigmented macules, angiofibromas, shagreen patches, fibrous plaque, and ungual fibromas, whereas renal lesions include angiomyolipomas, cysts, lymphangiomas, and renal cell carcinoma that can interfere with renal function causing hypertension and hemorrhage. Since the prevalence of renal manifestations increases with age, ultrasonographic follow-up is required every 1–3 years [23].

Nail-patella syndrome is an autosomal dominant condition affecting the nails, skeletal system, kidneys, and eyes, and while renal failure can appear later in life, the most common sign is having missing or underdeveloped fingernails and toenails [24].

Neurofibromatosis type I (NF1) is a genetic disorder due to a mutation in or deletion of the NF1 gene that produces neurofibromin, a tumor suppressor protein. NF1 is usually recognized by axillary freckling, café-au-lait spots, and neurofibromas, but the kidney may also be involved in developing WT or angiomyolipomas. This disease also affects the bones, eyes, and nervous system [25].

Beckwith-Wiedemann syndrome is an overgrowth disorder due to abnormalities involving genes on chromosome 11. Beckwith-Widemann syndrome is characterized by large kidneys with medullary dysplasia, renal cysts, and urinary tract anomalies, neuroblastoma or WT (approximately 7.5% of cases), hemihypertrophy, and facial nevus flammeus in skin [26].

Adams-Oliver syndrome 1 is a genetic disorder that manifests with occasional urinary tract anomalies associated with a more common aplasia cutis congenita of the parietal region, trunk or limbs, cutis marmorata, telangiectasia congenita, and hypopigmented skin [26].

Apert syndrome is an autosomal dominant disease, with onset in infancy, that presents with craniofacial anomalies, syndactyly, hyperhidrosis, and pronounced acne at adolescence. In 10% of cases, it is associated with polycystic kidneys and hydronephrosis [26].

Fanconi pancytopenia syndrome commonly affects the skin, producing brownish pigmentation, and the kidneys by developing different renal anomalies such as hypoplasia or malformation [26].

Pallister-Hall syndrome usually affects both kidneys with renal ectopia or dysplasia and the skin with midline facial hemangioma [26].

## Conclusions

We could not find any reports that focused attention on the link between nephrological and dermatological manifestations of the same disease in children. It is also important for clinicians to keep in mind that in what may appear to be an exclusively dermatological disease, there can be nephrological manifestations as part of the same disorder and vice versa. Whenever a kidney disease is suspected, the following tests must always be performed: urinalysis and quantification of proteinuria, serum creatinine, and urea and an ultrasound of kidneys.
